# The influence of rural clinical school experiences on medical students’ levels of interest in rural careers

**DOI:** 10.1186/1478-4491-12-48

**Published:** 2014-08-28

**Authors:** Vivian Isaac, Lisa Watts, Lesley Forster, Craig S McLachlan

**Affiliations:** Rural Clinical School, University of New South Wales, Sydney, Australia

**Keywords:** Rural career intention, Interest level, Australian Rural Clinical School (RCS)

## Abstract

**Background:**

Australian Rural Clinical School (RCS) programmes have been designed to create experiences that positively influence graduates to choose rural medical careers. Rural career intent is a categorical evaluation measure and has been used to assess the Australian RCS model. Predictors for rural medical career intent have been associated with extrinsic values such as students with a rural background. Intrinsic values such as personal interest have not been assessed with respect to rural career intent. In psychology, a predictor of the motivation or emotion for a specific career or career location is the level of interest. Our primary aims are to model over one year of Australian RCS training, change in self-reported interest for future rural career intent. Secondary aims are to model student factors associated with rural career intent while attending an RCS.

**Methods:**

The study participants were medical students enrolled in a RCS in the year 2013 at the University of New South Wales (UNSW) and who completed the newly developed self-administered UNSW Undergraduate Destinations Study (UDS) questionnaire. Data were collected at baseline and after one year of RCS training on preferred location for internship, work and intended specialty. Interest for graduate practice location (career intent) was assessed on a five-variable Likert scale at both baseline and at follow-up. A total of 165 students completed the UDS at baseline and 150 students after 1 year of follow-up.

**Results:**

Factors associated with intent to practise in a rural location were rural background (χ^2^ = 28.4, *P* < 0.001), two or more previous years at an RCS (χ^2^ = 9.0, *P* = 0.003), and preference for a rural internship (χ^2^ = 17.8, *P* < 0.001). At follow-up, 41% of participants who originally intended to work in a metropolitan location at baseline changed their preference and indicated a preference for a rural location. The level of interest in intended practice location was significantly higher for those intending to work in a rural area than those with intention to work in a metropolitan (urban area) location (*t* = -3.1, *P* = 0.002). Initial rural career location intention was associated with increased interest levels after 1 year of follow-up (paired *t* = -2.3, *P* = 0.02).

**Conclusion:**

When evaluating the success of RCS outcomes with respect to rural workforce destination, both rural practice intentions and level of interest are key factors related to projected career destination. RCS experience can positively influence practice intent (toward rural practice) and interest levels (toward greater interest in rural practice).

## Background

The shortage of medical practitioners in rural and regional areas is well documented in Australia [1]. There have been considerable efforts from the Australian Federal Government to address these shortages by funding a range of programmes aimed at medical students. These programmes aim to increase medical student’s level of motivation for rural practice via providing students with extended rural clinical placements [2, 3]. One such initiative has been the establishment of the Rural Clinical School (RCS) programme across all medical schools in Australia.

A rural career pathway has been defined in previous studies as the intention or actual practice in a rural area [4]. RCS evaluation measures for rural career intentions have traditionally used a categorical ‘Yes/No’ binary factor [5]. Cross-sectional studies on predictors of rural career intent have focused on individual extrinsic values such as rural background [6, 7]. On the other hand, it is not known if RCS career intentions are intrinsically stable. That is, with respect to emotion and/or motivation [8]. Indeed there may be change in intrinsic values during decision-making over time [9].

Individual intrinsic values such as interest have been shown to provide insight into both individual values concerning motivation and personal emotion in career choice [10, 11] Assessment of intrinsic values (that is interest for a specific career or career location) has been shown to be a strong predictor of actual career practice [12, 13]. In career counselling and human resources management, use of interest levels have been used as a research tool to better understand and direct career choice [12, 14]. However, with respect to RCS evaluation for rural career intent, interest level has not been previously examined over time.

The primary aims of the present study are: i) to model over one year of Australian RCS training, and determine if self-reported interest for future rural career intent may vary; ii) to identify factors associated with rural practice intentions for medical students attending a RCS programme as part of their undergraduate studies.

## Methods

The study was approved by the UNSW Ethics Committee; all students who participated provided informed written consent. All medical students enrolled in the RCS programme at the University of New South Wales (UNSW) in the year 2013 were invited to participate in the Undergraduate Destination Study (UDS) by completing a self-administered questionnaire (Table 1). Students can spend anywhere from one to four years in the RCS (see Table 1) [5]. Of all 176 students, 169 questionnaires were available for processing and analyses. Four international students were excluded, leaving a sample of 165 for analyses. The UNSW RCS programme and demographics has been previously described [5].

**Table 1:** **Key variable of interest in the Undergraduate Destination Survey (baseline and follow-up)**

Variables	Question
Rural/non-rural background	Through which entry programme did you enter UNSW Medicine?
Cohort	In what year are you currently studying?
Number of years at RCS	Is this your first, second, third or fourth year at the RCS?
Conscription status	Did you choose to study at the RCS?
Preferred internship location	At this point in time, what is your preferred location to do your internship? (Metropolitan, Outer Metro, Regional, Rural, Remote, Unsure)
Level of interest in internship location	How strong is your interest in doing your internship in that location?
	On a scale of 1 to 5, with 1 = not very strong at all, and 5 = very strong, please circle which applies to you.
Intended work location	When you have completed all your post graduate training, in what area do you intend to work? (Metropolitan, Outer Metro, Regional, Rural, Remote)
Level of interest in intended work location	How strong is your interest in working in that location (that is your answer to Q4)?
	On a scale of 1 to 5, with 1 = not very strong at all, and 5 = very strong, please circle which applies to you.
Specialty	At this point in time, in what area of medicine do you plan to work?

Abbreviations: RCS, Rural Clinical School; UNSW, University of New South Wales.

The UDS survey questionnaire was developed for the purpose of our RCS programme evaluation and has not been previously validated. The UDS also aims to prospectively investigate the changes in interest and rural career intentions of students during the RCS programme and the relationship to future career destination (once this becomes known). The questionnaire specifically assessed rural background entry details, preferred internship location, intended work location, choice of rural clinical training, and years in RCS (Table 1). Conscription is not assessed in the survey. Finally, we measured the strength of interest for the intended work location on a five-point Likert scale [15, 16]. The same Likert scale has previously been used to determine intrinsic interest [9].

Data were analyzed using the statistical package SPSS v. 21 (SPSS IBM, New York, NY, USA). Descriptive data were examined to determine study variables. Wald χ^2^ test was used to determine the factors associated for location intent, which is rural practice or metropolitan practice. The independent effect of variables after mutual adjustment was tested in a multivariate logistic regression model. Independent sample *t-*test was used to analyze the difference in level of interest in rural practice across student characteristics. Paired *t*-test and repeated measure analysis of variance (ANOVA) was applied to investigate the longitudinal changes in interest levels on rural practice intent.

## Results

The total sample consists of 165 student participants. One hundred and eighteen students exhibited intention to practise in a non-metropolitan location (regional, rural and remote), and of these respondents 49 indicated that they intended to work in a rural or remote area after graduation. Table 2 provides a summary of the characteristics of the sample population. Fifteen participants did not return the questionnaires at follow-up. These participants were not different with regard to their rural background and number of years of training at an RCS; however, they were less likely to have chosen RCS training (χ^2^ = 8.8, *P* = 0.007) or intended to work at rural setting at baseline (χ^2^ = 5.4, *P* = 0.03).

**Table 2:** **Population characteristics**

Characteristics		N = 165	%
Gender	Female	101	61.2%
	Male	64	38.8%
Entry programme	Local	48	29.1%
	Rural	117	70.9%
Number of years in RCS	One	65	39.4%
	Two	64	38.8%
	> Three	36	21.8%
Preference for RCS for clinical training	Yes	127	77.0%
	No	38	23.0%
Preferred location for intern	Metropolitan	59	35.8%
	Outer metro	10	6.1%
	Regional	41	24.8%
	Rural	32	19.4%
	Unsure	23	13.9%
Preferred location for work	Metropolitan	38	23.0%
	Outer metro	9	5.5%
	Regional	69	41.8%
	Rural	45	27.3%
	Remote	4	2.4%
Intended specialty	General practice	59	34.5%
	Paediatrics	14	8.5%
	Emergency	12	7.3%
	Surgery	20	12.1%
	Sub-specialty medicine	30	16.4%
	Anaesthetics/intensive care	7	4.2%
	Obstetrics/gynaecology	8	4.8%
	Psychiatry	2	1.4%
	Unsure	13	7.9%

Abbreviations: RCS, Rural Clinical School**.**

### Factors associated with work intentions in rural location

Table 3 provides a summary of the factors associated with work intentions for rural practice. Rural background (χ^2^ = 28.4, *P* < 0.001), two or more years spent in an RCS (χ^2^ = 9.0, *P* = 0.003), choice of rural placement (χ^2^ = 25.2, *P* < 0.001), preferred internship in a rural location compared to metropolitan location (χ^2^ = 17.8, *P* < 0.001) were associated with work intention in a rural location.

**Table 3:** **Factors associated with students’ intention to work in a rural setting**

		Work intention in a rural settings		Wald χ ^2^ (***P-***value)
		N	%	
Gender	Female	77	76.2%	
	Male	41	64.1%	2.9 (0.11)
Entry programme	Local	20	41.7%	
	Rural	98	83.8%	28.4 (<0.001)
Number of years in RCS	One	39	60.0%	
	Two	46	71.9%	2.1 (0.13)
	> Three	33	91.7%	9.0 (0.003)
Preference for RCS for clinical training	Yes	104	81.9%	
	No	14	36.8%	25.2 (<0.001)
Preferred location for intern	Metro/outer metro	37	54.4%	
	Regional/rural/remote	64	87.7%	17.8 (<0.001)
	Unsure	17	77.3%	2.8 (0.09)
Intended specialty	General practice	72	66.7%	
	Others	46	80.7%	3.5 (0.06)

Abbreviations: RCS, Rural Clinical School.

In the multivariate logistic regression rural entry, number of years in clinical training, that is > two years compared to one year of clinical training, preference for rural location for internship compared to metropolitan locations and choice of rural clinical training were associated with work intentions at a rural location.

### Level of interest among those with rural career intentions at baseline

**Figure 1:**
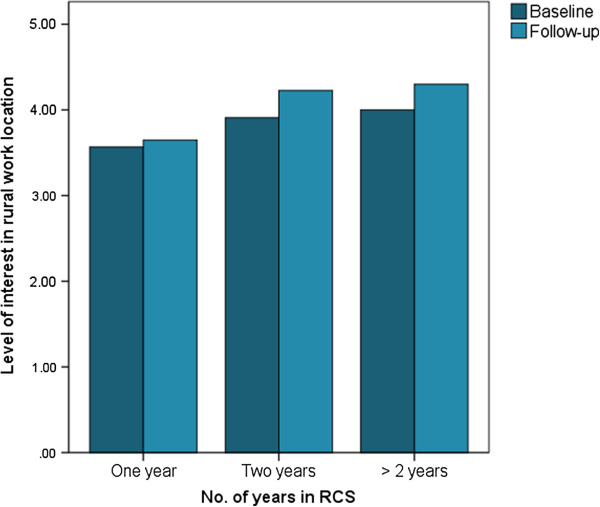
**Level of interest with number of years in Rural Clinical School (RCS) at baseline (only for students with rural intention, N = 118).**

Among participants with rural career intentions at baseline (N = 118), rural background and choice of rural placement were not associated with interest level. However, those participants who were ‘unsure’ on their internship location at baseline had lower interest levels compared to those others with either rural or metropolitan internship preference (*t* = -2.8, *P* = 0.006). More than one year in RCS showed a positive trend for higher interest levels in rural practice at baseline. The mean (SD) for one year RCS training was 3.6 (1.0) and two or more years RCS training was 4.0 (0.9) (Figure 1; Table 4).

**Table 4:** **Level of interest with number of years in Rural Clinical School (RCS) at baseline (only for students with rural intention, N = 118)**

	Level of interest in the intended work location					
	Baseline			Follow-up		
	N	Mean (SD)	F ( ***P-***value)	N	Mean (SD)	F ( ***P***-value)
One year	38	3.6 (1.0)		36	3.7 (1.0)	
Two years	46	3.9 (0.8)	2.2 (0.11)	44	4.2 (0.7)	6.7 (0.002)
Three or more years	33	4.0 (0.9)		30	4.3 (0.7)	

### Follow-up

Of the participants who originally intended to work in a metropolitan location on the baseline UDS, 41% changed their preference and indicated a preference for a rural location at the time of follow-up. Fifty-four point three percent of those who rated their rural interest less than 3 points increased to more than 4 points while only 10% had a drop in interest. Of the participants with rural work intention at baseline, 6.3% switched to metropolitan location after 1 year.

**Figure 2:**
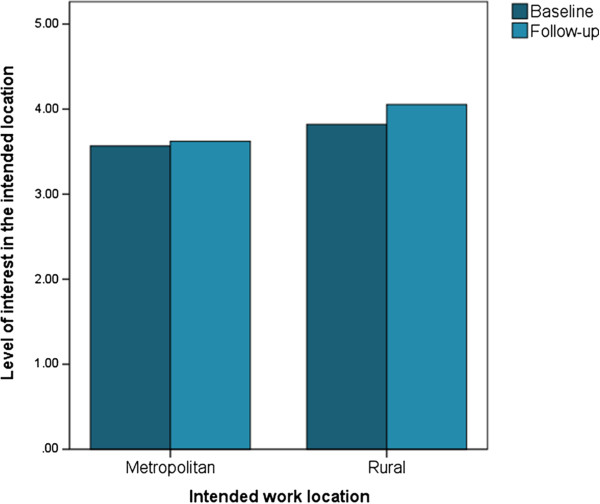
**Level of interest among those with rural and metropolitan work intentions at baseline.**

Level of interest was not statistically different between those with rural or metropolitan work intentions at baseline (*t* = -1.5, *P* = 0.12); however, at follow-up the level of interest in the intended location was significantly higher for those with rural intentions than metropolitan location at baseline (Figure 2; Table 5). Among those with rural intentions there was a slight but significant increase in the interest levels, the mean value at baseline was 3.8 (0.9) and the mean at follow-up was 4.1 (0.9) between baseline and after 1 year of RCS training (paired *t* = -2.3, *P* = 0.02) (Figure 2; Table 5). However, no significant interaction was seen with variables of interest and longitudinal change in interest level of rural practice.

**Table 5:** **Level of interest among those with rural and metropolitan work intentions at baseline**

	Level of interest in the intended work location						
	Baseline			Follow-up			Paired ***t***( ***P***-value)
	N	Mean (SD)	***t***( ***P***-value)	N	Mean (SD)	***t***( ***P***-value)	
Metropolitan	45	3.6 (1.0)	-1.5 (0.12)	39	3.6 (0.7)	-3.1 (0.002)	-0.15 (0.8)
Rural	118	3.8 (0.9)		111	4.1 (0.8)		-2.3 (0.02)

## Discussion

A significant number of studies have clearly evaluated the experience of training at RCS and its positive influence on a student’s intent for future rural training and practice [6, 17]. The Graduate Destination Study at UNSW also found there was a positive association between time spent at the RCS and rural career intention [5]. Our current study here suggests that number of years in RCS training not only has a positive association with students’ intention to work in a rural location, but also is positively associated with students’ level of interest in rural practice.

Our study also validates previously identified extrinsic factors associated with rural workforce intentions for rural medical students [6, 7]; that is students with a rural entry background and a greater length of training at a RCS had a greater intent for rural practice. Importantly, we also showed intrinsic values for rural work intentions related to stronger interest levels. Particularly, levels of interest increased over one year of follow-up for those students with rural work intentions. This suggests that a clinical training model of extended rural placements of more than one year increases interest in rural practice.

Interest level is an assessment tool that has been studied in vocational career selection. Particularly, it has been used to assess both career intent and also location. However, interest, for medical career location or specialty choice has received little attention [18]. In our study, rural work intentions and longer time spent in RCS were both associated with increased career location interest levels. Rural career intent and the positive experience in a novel environment could serve as an effective mechanism for cultivating competencies - self- efficacy that may subserve higher intrinsic interest levels [19].

From a behavioural perspective we suggest that a positive experience in a rural environment could reinforce rural motivations and explain why there was an increase in interest in rural medicine practice [20–23]. A reduction in interest levels in RCS students with a metropolitan career intent over time was noted in our study. We speculate this finding may have occurred because metropolitan intent students had a positive rural experience that eroded their intrinsic interest for metropolitan careers, despite maintaining a career intention for metropolitan practice. Indeed, studies suggest there can be extrinsic intrinsic conflict in decision-making when one’s environment is modified or positive experiences are derived from a work or study environment [11, 24, 25].

## Conclusion

The significance of our study is in demonstrating that interest level for an intended rural career in medicine increases over an academic year spent in a rural setting at a RCS. Level of interest could provide a valuable assessment tool for Rural Clinical School programme evaluation. Overall, we interpret our findings cautiously being a single centre study with small sample size and utilizing an evaluation survey tool designed internally. We also acknowledge extrinsic factors such as the limited availability of rural internships which are influential in graduates’ decisions about rural work locations [4]; however, this would unlikely diminish interest levels. Larger longitudinal studies are warranted to confirm the explanatory power of interest over and above rural intentions. As previous models have shown, that interest level is influenced by motivations, emotions and self-efficacy and these domains also deserve further exploration. Irrespective of these limitations, our study provides interest level as an additional measure of intended uptake of rural clinical practice after graduation.

## Authors’ information

Vivian Isaac is a PhD student at Rural Clinical School, UNSW. His main interests are evaluation methodology, population health research, cognitive neuroscience, quality of life and outcome research.

Lisa Watts is a research officer at the UNSW Rural Clinical School. Her main interests are education research, rural health and qualitative research methods.

Lesley Forster is Associate Dean of Rural Health and Head of School, at the UNSW Rural Clinical School. Her main interests are rural health, rural medical education and administration.

Craig McLachlan is an associate professor and Director of Research at the UNSW Rural Clinical School. Specific research interests include translational chronic health research and developing pathways for medical undergraduate research.

## Abbreviations

RCS: Rural Clinical School
UNSW: University of New South Wales.
